# Translation and Validation of the Premenstrual Assessment Form-Short Form Questionnaire in Hungarian

**DOI:** 10.1089/whr.2023.0107

**Published:** 2024-03-27

**Authors:** Olívia Dózsa-Juhász, Alexandra Makai, Viktória Prémusz, Pongrác Ács, Márta Hock

**Affiliations:** Faculty of Health Sciences, Institute of Physiotherapy and Sports Science, University of Pécs, Pécs, Hungary.

**Keywords:** premenstrual syndrome, Women's Health, Premenstrual Assessment Form-Short Form, PAF-SF, validation

## Abstract

**Introduction:**

Premenstrual symptoms, including premenstrual syndrome and its more severe form premenstrual dysphoric disorder, are a set of somatic and psychological symptoms that occur during the luteal phase of the menstrual cycle. Our research aimed to adapt the Hungarian version of the Premenstrual Assessment Form-Short Form (PAF-SF), a questionnaire suitable for assessing premenstrual symptoms, and to examine its reliability, validity, and applicability.

**Methods:**

The questionnaire was validated according to Beaton's six-step guidelines. Our sample consisted of 198 menstruating women, 50 of whom completed the instrument for a second time to assess reliability. Descriptive statistics were calculated presenting mean (standard deviation), the internal consistency was measured by Cronbach's alpha value, the test–retest reliability using intraclass correlation coefficients, Spearman rank correlation was applied to test the criterion validity of the questionnaire, and discriminant validity was examined using the independent-sample *t* test using IBM SPSS 28.0 software. The structural validity was evaluated by confirmatory factor analysis (CFA) using IBM AMOS 29.0 software. The level of significance was set at *p* < 0.05.

**Results:**

Our sample (average age 25.37 ± 4.80 years) scored 28.08 ± 9.49 points out of the maximum 60 points when filling out the PAF-SF questionnaire. The result of Cronbach's alpha calculation, which supports the reliability of the questionnaire, was 0.845. During the CFA, the three-factor structure (Affect, Water Retention, and Pain) was supported (root mean-square error approximation [RMSEA] = 0.054; Tucker–Lewis Index = 0.965; Comparative Fit Index = 0.976; *χ*^2^ = 48.642; df = 31; *p* = 0.023; *χ*^2^/df = 1.569).

**Discussion:**

The PAF-SF questionnaire proved to be a reliable measuring tool for assessing premenstrual symptoms among women of reproductive age.

## Introduction

Premenstrual symptoms, such as premenstrual syndrome (PMS) and its more severe variant premenstrual dysphoric disorder (PMDD), are a combination of physical and psychological symptoms that occur during the luteal phase of the menstrual cycle.^1^

Based on the latest data, half of women of reproductive age experience some level of premenstrual discomfort, of which 20%–30% have moderate to severe PMS symptoms, and another 3%–8% meet the PMDD criteria defined in The 5th Edition of the Diagnostic and Statistical Manual of Mental Disorders.^2^

Regarding the pathophysiology of PMS/PMDD, there is no specific position; the most likely theory ascribes a role to the fluctuation of sex hormones in the ovaries located on the axis of the hypothalamus–pituitary–sex organs. Changes in hormone levels often cannot be detected during a blood test, and rather it may be that the person in question shows an increased sensitivity to fluctuations in hormone levels.^2^

The diagnosis of PMS/PMDD requires a thorough medical history and physical examination, and it is common for the specialist to recommend keeping a diary of symptoms for at least two cycles. To be diagnosed with PMDD, the patient must have 5 out of 11 physical, behavioral, or cognitive-affective symptoms, and at least one of these must be a key symptom affecting mood. Such a symptom is, for example, irritability, mood swings, depressed mood, or anxiety. Regarding their temporal appearance, the symptoms are strongest 7–14 days before menstruation, weaken during the menstrual bleeding, and disappear completely in the follicular phase. These symptoms can be so strong that they can significantly affect the person's daily life and interpersonal relationships, and it is also important to emphasize that to make a diagnosis, it must be separated from the symptoms of other pathologies.^3^

There is currently no clinical test available to diagnose PMS/PMDD, so research related to the disease is hindered by the lack of appropriate diagnostic tools.^4^ That is why it is important to emphasize the professional validation and distribution of already existing, proven reliable tools (questionnaires).

The aim of our research was to translate the English version of the Premenstrual Assessment Form-Short Form (PAF-SF) into Hungarian, a questionnaire suitable for assessing premenstrual symptoms, and to examine its reliability, validity, and applicability.

## Materials and Methods

### Characteristics of the PAF-SF

The 95-item PAF originally developed by Halbreich et al. proved to be a reliable instrument.^5^ Still, over time it became necessary to create a shortened version, whose adaptation, validity, and reliability into English were examined by Allen et al. in 1991.^4^ The PAF-SF is a 10-item questionnaire developed to provide as much detailed information as possible about the various dysphoric premenstrual changes, whether about mood or physical condition. The questionnaire assesses these parameters through three subscales: (1) “Pain,” (2) “Water Retention,” and (3) “Affect.” The Affect subscale has four questions, while the Water Retention and Pain subscales have three questions each. The questions are scored on a 6-point scale ranging from 1 (no change) to 6 (extreme change), with the lowest possible score being 10 points and the highest score being 60 points and ask about symptoms during the last three menstrual cycles.

Regarding scoring, three tools are available to the professional: (1) specific criteria for categorical subtypes of premenstrual changes, (2) a summary of unipolar dimensional scales, and (3) dimensional measures of the bipolar continuum. Different ways of summarizing PAF data complement and provide an integrated and flexible approach to analyzing data from PAF.^4,5^ Lee et al. validated the PAF-SF questionnaire in the Korean language in 2002 and found it suitable for diagnosing PMS based on their research results; the diagnostic (cutoff) point limit for diagnosing moderate and severe PMS and PMDD was established at 27 points.^6^

### Presentation of the validation process

The original English questionnaire assessing premenstrual symptoms was translated into Hungarian and validated according to the six-step guidelines formulated by Beaton in 2000.^7^

As the very first step, we asked for the written permission and support of the author of the original English-language questionnaire to start the validation process. After all of this, the questionnaire was translated into Hungarian, which was completed by two independent persons: a health care worker with advanced English language examination and an independent person with a professional translator qualification. After that, the synthesis formed from the two Hungarian translations was translated back into English by two separate persons, one of whom was also qualified as a professional translator, and the other with a qualification as a health care worker and an advanced English language examination.

Finally, we considered the synthesis complete when the two independent translators gave written feedback that the questionnaire's content, form, and quality translated back into the original language showed no significant differences. After that, we carried out a pretest of the questionnaire involving 30 Hungarian women of reproductive age between 18 and 45 years. We corrected the possible interpretation difficulties they indicated, so the questionnaire that did not contain them was used for the internal consistency test. The internal consistency test was carried out with the involvement of 50 people who filled out the questionnaires twice during the test, exactly 2 weeks apart. After all this, we collected data with the completed, final version in Hungarian. For the external convergent validity test, the perceived stress level and mental state were assessed as external parameters in addition to the sociodemographic parameters and general and reproductive health questions.

*Presentation of the General Health Questionnaire-12 measurement tool:*

We employed Goldberg's 12-item General Health Questionnaire to gauge the presence of depression. The initial questionnaire comprised 60 questions, but various versions emerged as effective over time, including the 28-question and 12-question versions. All iterations of the questionnaire are designed to evaluate mental health across four subscales: somatic symptoms, anxiety and insomnia, social dysfunctions, and major depression. Participants responded to the 12 questions using a 4-point scale ranging from 0 to 3. When assessing the GHQ-12, two scoring systems can be utilized: bimodal (0-0-1-1) and Likert (0-1-2-3). The outcomes are derived from the total scores of the participants. For the bimodal scoring method, the diagnostic threshold is 2/3, and the maximum score achievable is 12. The Likert scale's diagnostic point is 8/9, and the maximum score is 36.^8,9^

*Presentation of the Perceived Stress Scale measuring tool:*

We utilized the validated Hungarian version of the Perceived Stress Scale to evaluate perceived stress. This questionnaire provides insights into the stress level experienced over the previous month. Comprising 10 questions, the respondents were able to respond on a 5-point scale, ranging from 0 to 4. Consequently, the minimum score attainable is 0, while the maximum is 40. During the assessment process, individuals can be categorized into three groups based on their total scores: 0–13 points indicate low perceived stress, 14–26 points suggest moderate perceived stress and 27–40 points signify a high level of perceived stress.^10,11^

The study was approved by the Regional and Institutional Research Ethics Committee of PTE-KK and registered under file number 9386-PTE 2022.

This research article was conducted according to the Standard for the Reporting of Diagnostic Accuracy Studies guidelines (Supplementary Data S1).

The study adhered to the principles of the Declaration of Helsinki, with subjects participating voluntarily, receiving full information before data collection, and providing written Declaration of Consent.

### Participants and procedure

**FIG. 1.**
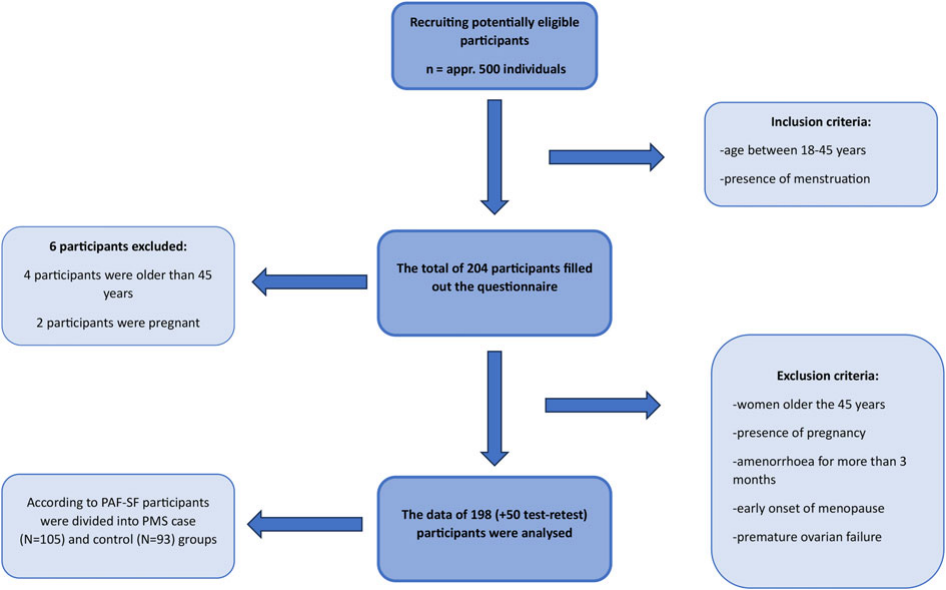
Representing the sampling process of the research.

For the cross-sectional questionnaire research, participants were recruited utilizing convenience sampling with the cooperation of the Faculty of Health Sciences of the University of Pécs and on the interface of various social networking sites (Fig. 1). The age of 18–45 and the presence of menstruation were defined as inclusion criteria in the research sample. In terms of exclusion criteria, women who are older than 45 years, who are pregnant, who have not menstruated for more than 3 months, and who suffer from early menopause or premature ovarian failure could not be included in the sample. Based on this, our sample was finally made up of 198 people. When determining the number of items, we considered the rule that there should be at least 10 times as many participants as the number of questions in the questionnaire intended for validation.

### Statistical analysis

We used the Microsoft Office Excel program (Microsoft Corporation, Redmond, WA) to create a database during the research. Then the statistical analyses were performed using IBM SPSS version 28.0 software and IBM SPSS AMOS 29.0 (SPSS, Inc., Chicago, IL) version software. We made a descriptive statistical analysis, and the data were expressed by determining mean ± standard deviation and frequency (%). The Kolmogorov–Smirnov test was used to test normality. While examining the reliability of the new questionnaire adapted to the Hungarian language, we calculated Cronbach's alpha value to determine the internal consistency of the measuring instrument.^12^ The test shows to what extent the individual items of the measuring device or subscales measure the same thing, and its value can vary between 0 and 1; in our study, we considered the test reliable between 0.5 and 0.95.^13^

During the test–retest study, intraclass correlation coefficients (ICCs),^14,15^ Wilcoxon test, and paired *t*-test were calculated.^16^ The validity of the questionnaire's factors was checked using confirmatory factor analysis (CFA). CFA is a structural equation modeling technique to determine the fit between a hypothesized factor structure and empirical data. It is generally accepted and recommended to judge the model's goodness of fit based on several criteria. One of the most frequently used fit indicators is the *χ*^2^-test measure, which can generally be considered acceptable if its value compared with the degree of freedom is low (*e.g.*, less than twice the degree of freedom) and not significant (*p* > 0.05).^17^

While there is no universal cutoff for fit indices, our study followed the following guidelines: close fit (root mean-square error approximation—RMSEA ≤0,05), adequate or acceptable fit (0,05 < RMSEA ≤0,08), and if the Comparative Fit Index (CFI) and the Tucker–Lewis Index (TLI) were greater than or equal to 0.09.^18^ To examine external convergent validity, Spearman's correlation analysis was performed between the premenstrual symptoms questionnaire and other variables such as perceived stress, mental state, reproductive health, and social and demographic factors.^19^ Independent-sample *T*-test was used for the discriminant validity test. The significance level was defined as *p* < 0.05.

## Results

One hundred ninety-eight women of reproductive age participated in our research, whose average age was 25.37 ± 4.80 years. The average total score of the PAF-SF was 28.08 ± 9.49 points out of a possible 60 points, while the average total score of the Perceived Stress Scale (PSS) was 19.61 ± 7.98 points out of a maximum of 40 points, and the average total score of the General Health Questionnaire 12-item version (GHQ-12) was 4.43 ± 3.58 points out of 12 possible points. Based on the scores of the answers to the PAF-SF questionnaire, 53% of the subjects (*n* = 105) were included in the case group. Data related to the reproductive health of the sample are illustrated in the following table (Table 1).

**Table 1.** Data Related to the Reproductive Health of the Sample (*n* = 198)

	Frequency (***n***)	Percentage distribution (%)	Cumulative percentage (%)
Do you have regular menstrual cycles?			
No	42	21.2	21.2
Yes	156	78.8	100.0
On a scale of 1–5, how heavy is your bleeding during your monthly cycle?			
1	5	2.5	2.5
2	21	10.6	13.1
3	92	46.5	59.6
4	65	32.8	92.4
5	15	7.6	100.0
How many days does your period last?			
3	9	4.5	4.5
4	36	18.2	22.7
5	71	35.9	58.6
6	51	25.8	84.3
7	28	14.1	98.5
8	2	1.0	99.5
12	1	0.5	100.0
Do you have midterm bleeding?			
No	176	88.9	88.9
Yes	22	11.1	100.0
Do pain and cramps accompany your menstrual bleeding?			
No	37	18.7	18.7
Yes	161	81.3	100.0
The severity of pains and cramps during your period on a scale of 0–10			
0	34	17.2	17.2
1	6	3.0	20.2
2	8	4.0	24.2
3	13	6.6	30.8
4	22	11.1	41.9
5	14	7.1	49.0
6	22	11.1	60.1
7	39	19.7	79.8
8	21	10.6	90.4
9	12	6.1	96.5
10	7	3.5	100.0
Have you ever given birth to a child?			
No	173	87.4	87.4
Yes	25	12.6	100.0
Have you ever had an abortion?			
No	190	96.0	96.0
Yes	8	4.0	100.0
Do you use or take hormonal contraceptives?			
No	146	73.7	73.7
Yes	52	26.3	100.0
Have you ever been diagnosed with PCOS?			
No	169	85.4	85.4
Yes	29	14.6	100.0

PCOS, polycystic ovary syndrome.

During the internal consistency test, apart from the question of the Affect subscale regarding compliance with everyday expectations, no significant differences (*p* ≥ 0.05) were found between the answers received during the first and second filling (Table 2). The result of the Cronbach's alpha calculation, which supports the reliability of the questionnaire, was 0.845, which suggests excellent reliability, and the result of the ICC measured during the reliability test was perfect (ICC = 0.969; 95% confidence interval = 0.945–0.982). During the convergent validity test, Spearman correlation analysis was performed using the Perceived Stress Scale, which assesses the perceived stress level, and the General Health Questionnaire, which examines the mental state. There was a positive, significant correlation for both factors, illustrated in Table 3.

**Table 2.** Table Illustrating the Results of the Test–Retest Examination (*n* = 50)

Subscale	Question	Test score (mean and SD)	Retest score (mean and SD)	Results (***p***)
Affect	Have outbursts of “irritability” or bad temper	3.24 ± 1.00	3.26 ± 1.07	0.835^a^
	Feel sad or blue	2.96 ± 1.14	3.00 ± 1.11	0.852^a^
	Feel under stress	3.32 ± 1.36	3.50 ± 1.33	0.154^a^
	Feel that “I just can’ cope” or am overwhelmed by ordinary demands	2.26 ± 1.29	2.58 ± 1.18	0.004^a^
Water Retention	Have edema, swelling, puffiness, or water retention	1.34 ± 0.80	1.48 ± 0.86	0.052^a^
	Feel bloated	2.96 ± 1.46	2.78 ± 1.22	0.191^a^
	Have weight gain	2.12 ± 1.24	2.18 ± 1.17	0.346^a^
Pain	Have relatively steady abdominal heaviness, discomfort, or pain	3.34 ± 1.53	3.32 ± 1.42	0.904^a^
	Tend to have backaches, joint or muscle pains, or stiffness	2.58 ± 1.39	2.46 ± 1.27	0.157^a^
	Have pain, tenderness, enlargement or swelling of breasts	2.98 ± 1.52	2.96 ± 1.48	0.796^a^
Total scores		27.10 ± 8.43	27.52 ± 8.20	0.311^b^

^a^ Wilcoxon test.

^b^ Paired samples *t*-test.

SD, standard deviation.

**Table 3.** Table Summarizing the Results of the External Convergent Validity Test (*n* = 198)

	GHQ-12	PSS
PAF-SF		
Correlation (rho)	0.485^**^	0.395^**^
Significance (*p*)	*p* < 0.001	*p* < 0.001
Frequency (*n*)	198	198

PAF-SF, Premenstrual Assessment Form-Short Form; GHQ-12, General Health Questionnaire 12-item version; PSS, Perceived Stress Scale.

During the examination of the differences between the groups (discriminant validity), a two-sample *T*-test was used, the results of which showed that there is a significant difference between the scores of the case and control groups based on the PAF-SF questionnaire (*F* = 4.132; *p* < 0.001). CFA on the sample used supported the three-factor structure, such as the Affect, Water Retention, and Pain subscales in the case of the PAF-SF questionnaire (RMSEA = 0.054; TLI = 0.965; CFI = 0.976; *χ*^2^ = 48.642; df = 31; *p* = 0.023; *χ*^2^/df = 1.569).

## Discussion

Our study aimed to adapt the PAF-SF, an English-language questionnaire that assesses premenstrual symptoms, into Hungarian and to examine its reliability and validity. Regarding the study sample, more than half of the participants in the research struggled with moderate or severe premenstrual symptoms during the data collection period. The average score of the PAF-SF was 28.08 ± 9.49 points, which can be compared with the result of previous research.^4^ The Cronbach's alpha value, which supports the reliability of the questionnaire, was 0.845. The results of the internal consistency test showed that, apart from one question, the answers given during the test–retest survey did not differ significantly, which is also supported by the results of previous studies.^4,6^

The responses received during the test–retest survey regarding the question of everyday expectations of the Affect subscale showed a significant difference (*p* < 0.004); however, this can be explained by the fact that during the 2 weeks between the two fillings, the university examination period began for most of the respondents, which could greatly influence their mood and their feelings about coping.^20–22^ During the discriminant validity test, the two-sample *T*-test showed a significant difference between the average scores of the PAF-SF case and control groups (*F* = 4.132; *p* < 0.001), a result supported by the results of an already mentioned Korean study.^6^ During the convergent validity test, a positive significant correlation was shown with the results of the PSS and GHQ-12 questionnaires, which are supported by results found in several international literature.^23–26^

### Implications for practice

The present research draws attention to the importance of diagnosing PMS, which is essential for a deeper understanding of women's and menstrual health. The authors believe that the PAF-SF is a suitable tool that requires further studies to confirm its applicability in clinical practice. Due to its brevity and conciseness, it can be easily used in everyday practice and can provide a solution for uncovering and remedying previously unknown or seemingly insignificant problems.

### Strengths and limitations

Among the strengths of the research, it can be mentioned that few tools for measuring or summarizing premenstrual symptoms in Hungarian have been validated so far. Among the limitations of the research, it is important to mention that the PAF-SF questionnaire has so far been validated in few languages, however, the authors hope that this research can once again draw attention to the effectiveness and reliability of this measuring instrument, which will make it more widespread and will lead to more and more research and publications proving its clinical effectiveness.

## Conclusions

Based on the results of our research, the Hungarian version of the PAF-SF proved to be a reliable, objective measurement tool for assessing and evaluating premenstrual symptoms in the female population of reproductive age.

## Supplementary Material

Supplemental data

## Abbreviations Used

CFA: confirmatory factor analysis
CFI: Comparative Fit Index
ICC: intraclass correlation coefficients
PAF-SF: Premenstrual Assessment Form-Short Form
PCOS: polycystic ovary syndrome
PMDD: premenstrual dysphoric disorder
PMS: premenstrual syndrome
RMSEA: root mean-square error approximation
SD: standard deviation
TLI: Tucker–Lewis Index

## References

[1] Appleton SM. Premenstrual syndrome: Evidence-based evaluation and treatment. Clin Obstet Gynecol 2018;61(1):52–61. 29298169 10.1097/GRF.0000000000000339

[2] Halbreich U, Borstein J, Pearlstein T. The prevalence, impairment, impact, and burden of premenstrual dysphoric disorder (PMS/PMDD). Psychoneuroendocrinology 2003;28:1–23. 10.1016/s0306-4530(03)00098-2 12892987

[3] American Psychiatric Association. Diagnostic and Statistical Manual of Mental Disorders. American Psychiatric Association: Washington, D.C.; 2013.

[4] Allen SS, McBride CM, Pirie PL. The shortened premenstrual assessment form. J Reprod Med 1991;36(11):769–772. 1765953

[5] Halbreich U, Endicott J, Nee J. Premenstrual depressive changes value of differentiation. Arch Gen Psychiatry 1983;40(5):535–542. 6682307 10.1001/archpsyc.1983.01790050061007

[6] Lee MH, Kim JW, Lee JH, et al. The standardization of the shortened premenstrual assessment form and applicability on the internet. J Korean Neuropsychiatr Assoc 2002;41(1):159–167.

[7] Beaton DE, Bombardier C, Guillemin F, et al. Guidelines for the process of cross-cultural adaptation of self-report measures. SPINE 2000;25(24):3186–3191. 11124735 10.1097/00007632-200012150-00014

[8] Makowska Z, Merecz D, Mościcka A, et al. The validity of general health questionnaires, GHQ-12 and GHQ-28, in mental health studies of working people. Int J Occup Med Environ Health 2002;15(4):353–362. 12608623

[9] Balajti I, Vokó Z, Ádány R, et al. A koherencia-érzés mérésére szolgáló rövidített kérdőív és a lelki egészség (GHQ-12) kérdőív magyar nyelvű változatainak validálása. Mentálhig És Pszichoszomatika 2008;8(2):147–161.

[10] Cohen S. Perceived stress in a probability sample of the Unites States. Soc Psychol Health 1988;31–67.

[11] Stauder A, Konkoly Thege B. Az Észlelt Stressz Kérdőív (PSS) Magyar Verziójának Jellemzői. Mentálhig És Pszichoszomatika 2006;7(3):203–216.

[12] Cronbach LJ. Coefficient alpha and the internal structure of tests. Psychomerika 1951;16:297–334.

[13] Taber KS. The use of Cronbach's alpha when developing and reporting research instruments in science education. Res Sci Educ 2018;48(6):1273–1296.

[14] Beckerman H, Roebroeck ME, Lankhorst GJ, et al. Smallest real difference, a link between reproducibility and responsiveness. Qual Life Res 2001;10(7):571–578. 11822790 10.1023/a:1013138911638

[15] Koo TK, Li MY. A guideline of selecting and reporting intraclass correlation coefficients for reliability research. J Chiropr Med 2016;15(2):155–163. 27330520 10.1016/j.jcm.2016.02.012 PMC4913118

[16] Van Alstine AW, Viswanathan S. Test–retest reliability of the multifocal photopic negative response. Doc Ophthalmol 2017;134(1):25–36. 28035520 10.1007/s10633-016-9569-3

[17] Ropovik I. A cautionary note on testing latent variable models. Front Psychol 2015;6. 26594192 10.3389/fpsyg.2015.01715 PMC4635201

[18] Brown TA. Confirmatory factor analysis for applied research. Guilford Press: New York; 2015.

[19] Carlson KD, Herdman AO. Understanding the impact of convergent validity on research results. Organ Res Methods 2012;15(1):17–32.

[20] Worku D, Dirriba AB, Wordofa B, et al. Perceived stress, depression, and associated factors among undergraduate health science students at Arsi University in 2019 in Oromia, Ethiopia. Psychiatry J 2020;2020:1–8. 10.1155/2020/4956234 PMC7275200 32550225

[21] Acikgoz A, Dayi A, Binbay T. Prevalence of premenstrual syndrome and its relationship to depressive symptoms in first-year university students. Saudi Med J 2017;38(11):1125–1131. 29114701 10.15537/smj.2017.11.20526 PMC5767616

[22] Mohebbi M, Amir Ali Akbari S, Mahmodi Z, et al. Comparison between the lifestyles of university students with and without premenstrual syndromes. Electron Physician 2017;9(6):4489–4496. 28848621 10.19082/4489 PMC5557126

[23] Alshdaifat E, Absy N, Sindiani A, et al. Premenstrual syndrome and its association with perceived stress: The experience of medical students in Jordan. Int J Womens Health 2022;14:777–785. 35726296 10.2147/IJWH.S361964 PMC9206437

[24] Hou L, Zhou R. Patterns of premenstrual syndrome and depression symptoms in Chinese female university students: Results of a latent profile analysis. J Affect Disord 2021;293:64–70. 34174472 10.1016/j.jad.2021.06.017

[25] Kappen M, Raeymakers S, Weyers S, et al. Stress and rumination in Premenstrual Syndrome (PMS): Identifying stable and menstrual cycle-related differences in PMS symptom severity. J Affect Disord 2022;319:580–588. 36162688 10.1016/j.jad.2022.09.052

[26] Rafique N, Al-Sheikh MH. Prevalence of menstrual problems and their association with psychological stress in young female students studying health sciences. Saudi Med J 2018;39(1):67–73. 29332111 10.15537/smj.2018.1.21438 PMC5885123

